# Plant Cell Culture-Derived Saponin Adjuvant Enhances Immune Response Against a Stabilized Human Metapneumovirus Pre-Fusion Vaccine Candidate

**DOI:** 10.3390/vaccines12121435

**Published:** 2024-12-20

**Authors:** Maarten Swart, Jessica Allen, Brendan Reed, Ana Izquierdo Gil, Johan Verspuij, Sonja Schmit-Tillemans, Anish Chakkumkal, Mark Findeis, Angela V. Hafner, Chandresh Harjivan, Rebecca Kurnat, Harmjan Kuipers, Roland Zahn, Boerries Brandenburg

**Affiliations:** 1 Johnson & Johnson, Janssen Vaccines & Prevention, 2333 CN Leiden, The Netherlandsjverspui@its.jnj.com (J.V.); sschmit@its.jnj.com (S.S.-T.); achakkum@its.jnj.com (A.C.); harmjan.kuipers@gmail.com (H.K.); rzahn@its.jnj.com (R.Z.); 2 SaponiQx, 3 Forbes Road, Lexington, MA 02421, USAmark.findeis@agenusbio.com (M.F.); angela.hafner@saponiqx.com (A.V.H.); charjivan@gmail.com (C.H.); rebecca.kurnat@saponiqx.com (R.K.); 3 Agenus Inc., 3 Forbes Road, Lexington, MA 02421, USA

**Keywords:** human metapneumovirus, HMPV, Pneumoviridae family viruses, subunit vaccine, adjuvant, QS-21, cpcQS-21, Saponin, AS01

## Abstract

Human metapneumovirus (HMPV) is a significant respiratory pathogen, particularly in vulnerable populations. Background: No vaccine for the prevention of HMPV is currently licensed, although several subunit vaccines are in development. Saponin-based adjuvant systems (AS), including QS-21, have transformed the field of subunit vaccines by dramatically increasing their potency and efficacy, leading to the development of several licensed vaccines. However, naturally sourced tree bark-extracted QS-21 faces supply and manufacturing challenges, hindering vaccine development. Objective: This study reports on an alternative plant cell culture system for the consistent production of highly pure QS-21. Method: We evaluated the efficacy of cultured plant cell (cpc)-produced QS-21 in a novel HMPV vaccine, formulating a recombinant pre-fusion stabilized HMPV F protein (preF) with cpcQS-21 and a synthetic toll-like receptor 4 (TLR4) agonist adjuvant formulation. Results: In mice, TLR4 agonist containing adjuvant formulations with plant cell-produced QS-21 performed equally to licensed adjuvant AS01 containing tree-bark-extracted QS-21 and demonstrated a significant increase in immunogenicity against HMPV preF compared to the unadjuvanted control. Conclusion: Our findings pave the way for a reliable, scalable, and sustainable source of pure QS-21, enabling the development of highly effective HMPV and other vaccines with significant public health impact.

## 1. Introduction

Human metapneumovirus (HMPV), a negative-sense single-stranded RNA virus, belongs to the *Pneumoviridae* family, which also includes respiratory syncytial virus (RSV) and parainfluenza viruses [1]. HMPV primarily causes upper and lower respiratory tract infections in young children, older adults, and immunocompromised individuals [2]. In 2018, HMPV was responsible for an estimated 14.2 million acute lower respiratory infections in children under five years of age worldwide [3]. Thus, HMPV represents a major public health concern, and no licensed vaccine is currently available [4,5].

Many successful vaccines with a durable, antigen-specific response rely on potent, immunostimulatory adjuvant systems [6]. Vaccine adjuvants are crucial for enhancing the immune response’s magnitude, breadth, and durability to an antigen. Further, adjuvants can increase vaccine immunogenicity in target populations, such as neonates, the elderly, and immunocompromised individuals, thereby expanding vaccine utility and coverage [6,7]. Among the most potent adjuvants used in licensed vaccines are those formulated with the saponin QS-21 [8,9]. Until now, QS-21 has been extracted from the bark of the Chilean soapbark tree (beQS-21), *Quillaja saponaria* Molina, through a complex and laborious multi-step isolation process [10]. Due to the limited supply and projected demand for QS-21, sustainable methods to produce QS-21 are needed. Demand for QS-21 extends beyond the pharmaceutical industry, with competition from agriculture, cosmetics, and the food and beverage industries. Recently, a sustainable culture system derived from *Q. saponaria* plant cells has been established using SaponiQx, which produces saponin extracts with similar biochemical, biological, and immune stimulatory properties as compared to those isolated from tree bark [11]. Several cell lines have been developed from this culture system, each exhibiting slight variations in their saponin profiles. However, each individual line consistently produces the same composition of saponins [11]. The cell lines chosen for production were those that yielded the highest amounts of QS-21. The plant cell culture-derived QS-21 (cpcQS-21) was isolated with high purity and exhibited the same biological potency as the beQS-21 present in GlaxoSmithKline’s (GSK) adjuvant AS01 [11]. This process is currently being scaled up and is a promising solution to meet the demand for QS-21, which is crucial for developing novel adjuvant systems for future vaccines.

An example of a successful licensed adjuvant system that contains beQS-21 is AS01. AS01 is a liposomal formulation that contains beQS-21 and a toll-like Receptor 4 (TLR4) agonist, 3-*O*-desacyl-4′-monophosphoryl lipid A (MPLA), which is a purified derivative of lipid A from *Salmonella enterica* serovar Minnesota [4,7,8]. When administered individually, these immunostimulatory molecules induce suboptimal immune responses; however, when combined, they synergize and drive the production of antigen-specific antibodies, CD8+ T cells, and type 1 helper (Th1)-polarized response [12]. AS01 adjuvant is a critical component of several licensed vaccines, such as Shingrix^®^, Mosquirix^®^, and Arexvy^®^, and these vaccines have demonstrated high efficacy in clinical trials [13,14,15].

Given the success of AS01 as a vaccine adjuvant for a *Pneumoviridae* family virus (i.e., RSV, Arexvy^®^), we sought to evaluate the immunogenicity of an AS01-like adjuvanted vaccine for HMPV. Here, we evaluated the HMPV-specific humoral and T cell-mediated immune (T-CMI) responses of a novel recombinant protein vaccine containing the soluble pre-fusion conformation stabilized HMPV fusion protein (preF) [16] adjuvanted with cpcQS-21 and PHAD^®^, a synthetic TLR4 agonist, or AS01. This study aims to determine if an AS01-like adjuvant formulation containing cpcQS-21 (AS01-SPQX) exhibits adjuvant properties equivalent to commercial AS01, which contains QS-21 (AS01). We demonstrated that HMPV preF combined with AS01-SPQX or AS01 induced high anti-HMPV preF antibody titers and modest T-CMI responses. Importantly, the immune responses generated from AS01 comprising cpcQS-21 and beQS-21 were biologically equivalent. The results of this study provide a proof-of-concept for the use of cpcQS-21 and PHAD in vaccine adjuvant formulations and provide another example of an antigen-AS01 pairing as a promising vaccine candidate.

## 2. Materials and Methods

### 2.1. Animals and Ethics Statements

Mouse experiments were approved by the Dutch Central Authority for Scientific Procedures on Animals (Centrale Commissie Dierproeven) and conducted in accordance with the European guidelines (EU directive on animal testing 2010/63/EU and ETS 123) and local Dutch legislation. Eight-week-old female BALB/cAnNCrl mice were purchased from Charles River Laboratories (Sulzfeld, Germany ).

### 2.2. Immunization and Sample Collection

Five mice per treatment group were vaccinated intramuscularly (i.m.) in the hind leg on days 0 and 28 (50 µL in the left and right hind leg, respectively). The vaccine was prepared by combining 5 µg of recombinant HMPV preF protein [16] with or without adjuvant formulations (Table 1). Blood was collected on day 27 and day 42 by submandibular vein and cardiac puncture, respectively. On day 42, animals were euthanized via cardiac puncture and cervical dislocation, and the spleens were collected. Body temperatures were measured every 24 hours between 4 days prior and 6 days post-vaccination using IPTT-300 Temperature Transponders (Plexx, Elst, The Netherlands).

### 2.3. Antibody Measurement

The serum was isolated from the blood, and HMPV F-specific IgG antibodies were measured using an enzyme-linked immunosorbent assay (ELISA). Briefly, 96-well ½ area, high-binding Optiplates (Revvity, Groningen, The Netherlands) were coated with streptavidin (Thermo Fisher Scientific, Bleiswijk, The Netherlands) at 0.25 µg/well and incubated for 2 h at 37 °C. Plates were washed with phosphate-buffered saline (PBS) containing 0.05% Tween 20 (*v*/*v* PBS/0.05% Tween20) and subsequently blocked with Blocker™ Casein (Thermo Fisher Scientific). After incubation for 1 h at room temperature (RT), the plates were washed with PBS/0.05% Tween20 and then incubated with 0.075 µg/mL biotinylated HMPV preF protein for 1 h at room temperature. The plates were then washed with PBS/0.05% Tween20. Serum samples were serially diluted in PBS/0.05% Tween20, transferred to blocked ELISA plates, and incubated for 1 h at room temperature. After washing with PBS/0.05% Tween20, horseradish peroxidase (HRP)-labeled anti-mouse IgG (Bio-Rad, Veenendaal, The Netherlands) was added to wells at a 1:10,000 dilution and incubated for 1 h at room temperature. After washing, the wells were developed with Enhanced Chemiluminescence (ECL) substrate (Bio-Rad), added for 10 min at room temperature. The luminescence signal was measured using a Synergy Neo (Agilent BioTek, Middelburg, The Netherlands). The relative potency (RP) was calculated based on a reference serum pool obtained from BALB/c mice immunized twice with AS01 adjuvanted HMPV preF protein and included on every plate. The lower limit of detection (LLOD) was based on a 99% quantile of serum samples from control animals analyzed in this ELISA.

### 2.4. Measurement of T Cell Responses

The frequency of IFN-γ-secreting cells was quantified using a mouse IFN-γ ELISpot PLUS kit ALP (Mabtech, Nacka Strand, Sweden) according to the manufacturer’s instructions. Spleens were isolated and processed into single-cell suspensions using the gentleMACS™ Dissociator system (Miltenyi Biotec, Leiden, The Netherlands), after which red blood cells were lysed using Ammonium–Chloride–Potassium (ACK) lysis buffer (Lonza, Geleen, The Netherlands). Cells were washed in PBS, resuspended in medium (500 mL RPMI1640 (Gibco, Thermo Fisher Scientific) supplemented with 50 mL heat-inactivated FBS (HyClone Cytiva, Marlborough, MA, USA), 5 mL penicillin/streptavidin (Gibco), 5 mL MEM non-essential amino acids (Gibco), 132 µL 2-Mercaptoethanol (Gibco)), and seeded on anti-IFNy antibody pre-coated plates at a concentration of 5 × 10^5^ cells per well. Splenocytes were stimulated with either cell culture medium with DMSO (negative control), 1 µg/mL HMPV A2 F peptide pool (JPT), or 1 ng/mL phorbol 12-myristate 13-acetate (PMA) and 1 µg/mL ionomycin (positive control) for 18 hours at 37 °C. Spots were enumerated, and analysis was performed using the A.EL.VIS Eli.Scan EliSpot Scanner and Eli.Analyse V6.1 (both Active Bioscience GmbH, Hamburg, Germany). Spot-forming units (SFUs) per 1 × 10^6^ splenocytes were calculated. The LLOD was based on the 95th percentile of the background response in the wells stimulated with cell culture medium plus DMSO.

### 2.5. QS-21 Liposome Formulations

Commercial AS01(B) was used as a benchmark formulation, containing 100 μg MPLA and 100 μg QS-21 in 500 μL produced by GSK with beQS-21 (QS Molina, fraction 21) (GlaxoSmithKline Biologicals, Rixensart, Belgium). cpcQS-21 was extracted using a sustainable cell culture system derived from *Q. Saponaria* plant cells that was established using SaponiQx (Lexington, MA, USA) and produces saponin extracts with similar biochemical, biological, and immune stimulatory properties as compared to those isolated from tree bark [11]. The manufacturing process used for producing large quantities of saponin-based adjuvant using *Q. Saponaria* plant cell culture is explained in detail in a previous study [11]. A comprehensive analytical characterization of a representative batch of cpcQS-21 used in this study was compared-21, to confirm their equivalency [11]. cpcQS-21-containing liposome formulations, AS01-SPQX and AS100-SPQX, were generated by Avanti Polar Lipids (Alabaster, AL, USA) using cpcQS-21 (Agenus and SaponiQx, Lexington, MA, USA). AS01-SPQX consisted of 100 µg/mL cpcQS-21, 1 mg/mL DOPC (Avanti Polar Lipids), 263 µg/mL cholesterol (Avanti Polar Lipids), 100 µg/mL PHAD^®^ (Avanti Polar Lipids). AS100-SPQX consists of 100 µg/mL cpcQS-21, 1 mg/mL DOPC (Avanti Polar Lipids), 295 µg/mL Cholesterol (Avanti Polar Lipids), 40.0 µg/mL PHAD^®^ (Avanti Polar Lipids).

**Table 1:** Adjuvant formulations.

Formulation Name	Source of QS-21	QS-21 μg/mL	TLR4 Agonist	TRL4 Agonist μg/mL
AS01	Bark extract from GSK	100	MPLA	100
AS01-SPQX	Cell culture from SaponiQx	100	PHAD^®^	100
AS100-SPQX	Cell culture from SaponiQx	100	PHAD^®^	40

### 2.6. Statistical Analysis

The responses between adjuvant formulations were compared across doses by ANOVA with adjuvant and dose as factors. The comparisons between AS100-SPQX and AS01-SPQX high dose or AS01 high dose were performed by a t-test from an ANOVA with the treatment group as a factor. The non-parametric Cochran-Mantel-Haenszel test or Mann-Whitney U-test were used if a group had more than 50% censored measurements or a normal distribution could not be assumed. A *p*-value equal to or below 0.05 was considered significant. All statistical analyses were performed using SAS (Cary, NC, USA).

## 3. Results

### 3.1. AS01 Formulated with cpcQS-21 or beQS-21 Elicited Similar Humoral Immune Responses in a Prime-Boost Model of HMPV Vaccination

**Figure 1:**
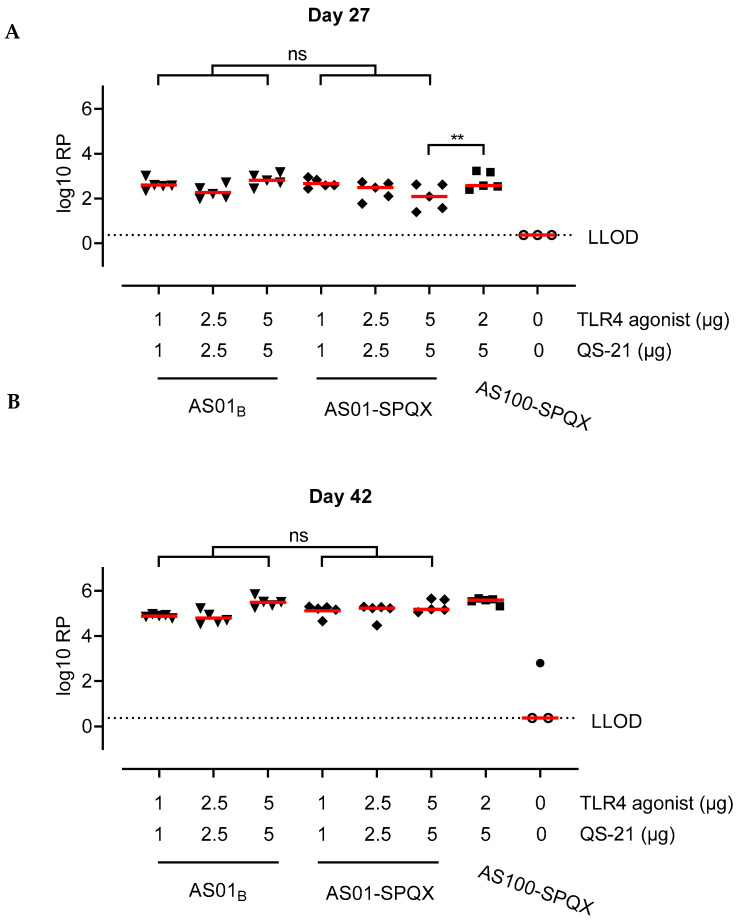
Anti-F antibody titers following immunization. AS01 formulated with cpcQS-21 or beQS-21 elicited similar humoral immune responses in a prime-boost model of HMPV vaccination. Anti-F serum IgG response from BALB/c mice immunized with two doses (day 0 and 28) of 5 µg recombinant HMPV A2 PreF combined with AS01_B_, AS01-SPQX, or AS100-SPQX. Anti-F antibody titers were measured by ELISA on day 27 (**A**) or 42 (**B**). Concentrations of TLR4 agonist (MPLA for AS01_B_ or PHAD^®^ for SPQX) and (be or cpc) QS-21 are indicated. Log10 relative potency (RP) titers are compared with a reference serum pool. Red horizontal bars indicate the median response per group, and the dotted line indicates the lower limit of detection (LLOD). Open symbols indicate that the response is at or below the LLOD. AS01B and AS01-SPQX were compared across doses by a t-test. AS100-SPQX was compared with 5:5 μg AS01B and 5:5 μg AS01-SPQX by a *t*-test. (ns, not significant; ** *p* ≤ 0.01).

BALB/c mice (*n* = 5) received two intramuscular doses of 5 µg soluble recombinant HMPV preF or preF co-formulated in liposomes containing beQS-21 and MPLA (AS01) or cpcQS-21 and PHAD^®^ (AS01-SPQX or AS100-SPQX, Table 1). To evaluate the potency and reactogenicity of different AS01 and SPQX dosages and ratios, mice received formulations containing 1, 2, 2.5, or 5 μg of synthetic TLR4 agonist PHAD^®^ and QS-21. Animals receiving unadjuvanted preF had no detectable levels of anti-F antibody titers. However, compared to immunization with unadjuvanted preF, all adjuvanted formulations elicited significantly higher antibody titers after one immunization (day 27) (Figure 1A). A second immunization boosted anti-F titers over 100-fold compared to one immunization (Figure 1B). Compared to mice that received unadjuvanted preF, all adjuvant groups induced significantly higher anti-F IgG titers after two doses of vaccine (Figure 1B). There were no significant differences in anti-F IgG titers between mice receiving HMPV preF with commercial AS01, AS01-SPQX, or AS100-SPQX.

As a surrogate for adjuvant reactogenicity, body temperatures were measured post-immunization, and no significant changes in body temperatures were observed throughout the study (Figure S1).

### 3.2. HMPV Vaccine Formulations Containing cpcQS-21 or beQS-21 Induced HMPV-Specific T Cell-Mediated Immunity

**Figure 2:**
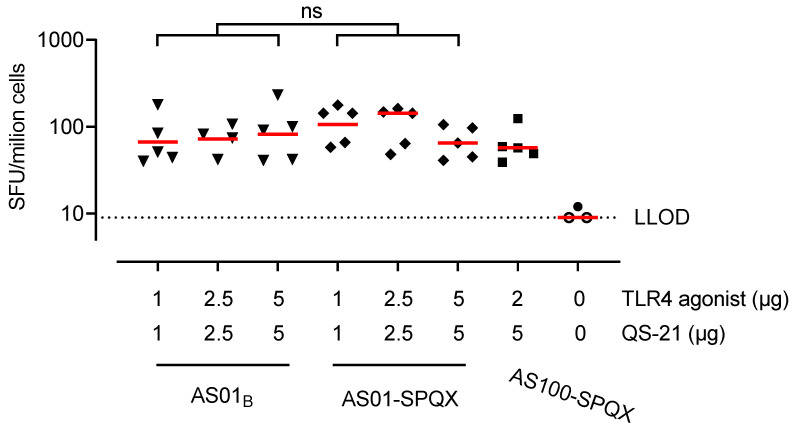
IFN-γ-secretion by splenocytes stimulated with an HMPV A2 F peptide pool. HMPV vaccine formulations containing cpcQS-21 or beQS-21 induced HMPV-specific T cell-mediated immunity. Fourteen days following the second immunization (boost), IFN-γ secretion was assessed upon ex vivo stimulation with an F-pool for 18 h. The frequency of IFN-γ-secreting cells is depicted as the number of spot-forming units (SFU) per million splenocytes. Horizontal red bars denote group geometric means, and horizontal dashed lines indicate the lower limit of detection (LLOD) based on the 95th percentile of the background response. Open symbols indicate the response is at or below the LLOD. AS01B and AS01-SPQX adjuvant formulations were compared across doses by a Cochran–Mantel–Haenszel test. AS100-SPQX was compared with 5:5 μg AS01B and 5:5 μg AS01-SPQX with a Mann-Whitney U-test. No significant differences were found. ns, not significant.

To assess the HMPV-specific T cell response following immunization, splenocytes from immunized mice were stimulated ex vivo with an HMPV A2 F peptide pool on day 42. IFN-γ production was determined by ELISpot and was largely undetectable in mice that received unadjuvanted preF protein (Figure 2). In contrast, all AS01, AS01-SPQX, and AS100-SPQX formulations induced significantly higher IFN-γ levels than immunization with unadjuvanted preF protein (Figure 2).

## 4. Discussion

A highly efficacious human metapneumovirus (HMPV) vaccine remains a critical public health priority, particularly for vulnerable populations, such as infants, the elderly, and immunocompromised individuals [2]. While several HMPV vaccine candidates have been evaluated in clinical trials, their efficacy has been limited [17,18]. In this study, we investigated the immunogenicity of a novel pre-fusion stabilized HMPV F protein [16] adjuvanted with AS01-like formulations containing either tree-bark extracted (be) or cultured plant cell (cpc) produced QS-21.

Our findings demonstrate the potential of cpcQS-21 as a viable and sustainable alternative to beQS-21. While plant cell culture systems may have inherent limitations in terms of growth rate compared to other expression systems, they offer significant advantages in scalability, reduced environmental impact, and consistent product quality [19]. Our data highlight the versatility of cpcQS-21 as a potent adjuvant for enhancing vaccine immunogenicity. We demonstrate that beQS-21- and cpcQS-21-containing adjuvants formulated with different TLR4 agonists induced robust humoral and cellular immune responses against HMPV preF characterized by high levels of anti-F IgG and IFN-γ production.

Of particular importance, both the humoral and cellular immune compartments were efficiently targeted with this vaccine, which is promising given the challenges in developing protective HMPV vaccines. The T cell compartment is thought to be critical for efficient clearance and resolution of HMPV infection, as immunocompromised and HIV-infected patients have a higher incidence of severe HMPV-related disease [20,21]. Recent studies have identified novel T cell epitopes that enhance viral clearance in animal models and are recognized by human T cells, highlighting the potential of T cell-based approaches in developing more effective and broadly protective HMPV vaccines [22]. Splenic IFN-γ production was observed from animals immunized with HMPV preF protein adjuvanted with AS01 or AS01/AS100-SPQX, indicative of a Th1 polarized immune response. Importantly, there were no significant differences in IFN-γ production among mice that received AS01 or AS01/AS100-SPQX, indicating that beQS-21 and cpcQS-21 induce similar cellular immune responses. Given the adjuvanted preF HMPV vaccine’s ability to elicit robust anti-F IgG and T cell-mediated responses, future studies will evaluate the vaccine’s resilience and efficacy in combatting viral challenges. This aligns with work by Lv et al., where immunization with varicella-zoster virus glycoprotein E (gE) antigen and AS01 or AS01-SPQX induce equivalent IgG and T cell-mediated responses [11].

Saponin-based adjuvant systems are regarded as one of the most efficacious options for protein subunit-based vaccines, and several vaccine candidates containing QS-21 are in development to target other infectious diseases, including HIV, SARS-CoV2, and malaria. However, naturally sourced QS-21 is limited and poses a barrier to vaccine production on a global scale. Thus, we have embraced a recently developed plant cell culture system that sustainably produces QS-21 with chemical and biological properties similar to bark-extracted QS-21 [11]. The study herein provides an additional example of how cpcQS-21 can successfully be paired with vaccine antigens to increase their immunogenicity. Testing adjuvants with various antigens is crucial for determining their broad applicability and effectiveness across different vaccines [23]. Research has demonstrated that adjuvants can vary significantly in their ability to enhance humoral and cellular immune responses when combined with different antigens [24]. Future research should broaden the comparison of adjuvants that contain QS-21 derived from plant tissues or cells [11], those biosynthesized using alternative organism culture technologies [25], and those that are chemically synthesized [26]. Additionally, further studies are necessary to assess the long-term protective efficacy of this HMPV vaccine candidate in relevant animal models and, ultimately, in human clinical trials.

## 5. Conclusions

This study demonstrates the promising immunogenicity of a novel pre-fusion stabilized HMPV F protein adjuvanted with a plant cell culture-derived saponin (cpcQS-21). The potent induction of both humoral and cellular immune responses highlights the potential of this adjuvant and vaccine candidate to provide effective protection against HMPV infection. However, further evaluation in relevant animal models and subsequent human clinical trials is necessary to fully assess its long-term efficacy and safety.

## Data Availability

All data are available in the article.

## Supplementary Materials

The following supporting information can be downloaded at: https://www.mdpi.com/article/10.3390/vaccines12121435/s1, Figure S1: Similar body temperature profiles for mice immunized with HMPV preF adjuvanted with cpcQS-21 or beQS-21 formulations.
