# Supplementary material for: Plant Cell Culture-Derived Saponin Adjuvant Enhances Immune Response Against a Stabilized Human Metapneumovirus Pre-Fusion Vaccine Candidate
Source: Vaccines (Basel). 2024 Dec 20;12(12):1435. doi: 10.3390/vaccines12121435 (PMC11728754; doi:10.3390/vaccines12121435)
Supplement: Supplementary file 1 [file vaccines-12-01435-s001.zip › vaccines-3288983-supplementary.pdf]

vaccines

MDPI

Supplemental Information

Plant cell culture-derived saponin adjuvant enhances the immune response against a stabilized human metapneumovirus pre-fusion vaccine candidate

Maarten Swart[1], Jessica Allen[2], Brendan Reed[2], Ana Izquierdo Gil[1], Johan Verspuij[1], Sonja Schmit-Tillemans[1], Anish Chakkumkal[1], Mark Findeis[2], Angela V. Hafner[2,3], Chandresh Harjivan[2], Rebecca Kurnat[2], Harmjan Kuipers[1], Roland Zahn[1], Boerries Brandenburg[1]*

1 Johnson & Johnson, Janssen Vaccines & Prevention, Leiden, The Netherlands
2 SaponiQx, Lexington, MA
3 Agenus Inc, Lexington, MA, USA.

* Correspondence: bbrande1@its.jnj.com

Supplemental Information

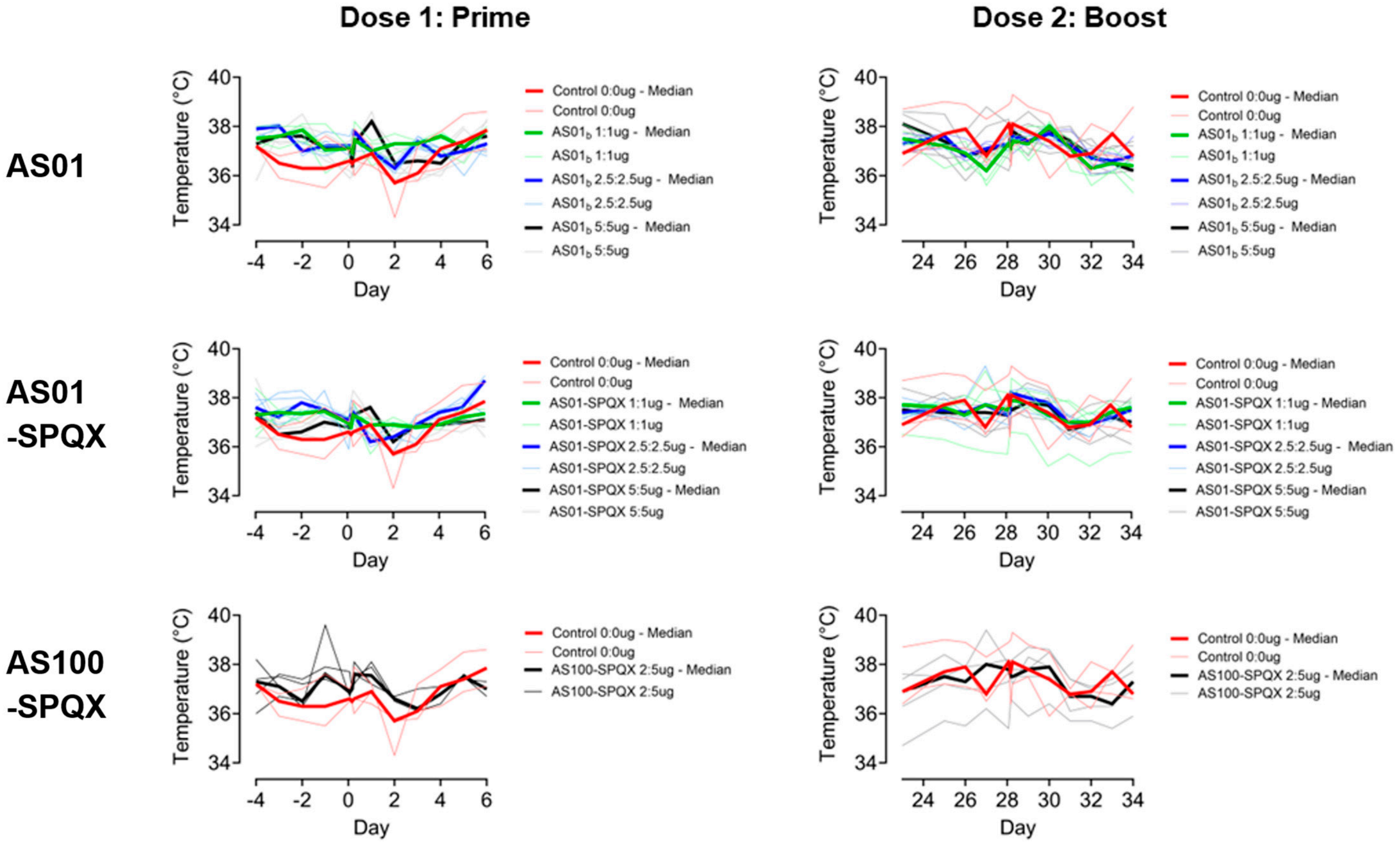

Supplemental Figure S1. Similar body temperature profiles for mice immunized with HMPV preF adjuvanted with cpcQS-21 or beQS-21 formulations. Body temperature of mice following immunization with HMPV preF adjuvanted with cpcQS-21 or beQS-21 formulations. Body temperature of BALB/c mice, as a surrogate for vaccine reactogenicity, was assessed before and after dose 1 (prime) and dose 2 (boost) of the HMPV vaccine formulations. Bold lines indicate group medians.
